# Classification of Extrapulmonary Manifestations Due to *Mycoplasma pneumoniae* Infection on the Basis of Possible Pathogenesis

**DOI:** 10.3389/fmicb.2016.00023

**Published:** 2016-01-28

**Authors:** Mitsuo Narita

**Affiliations:** Department of Pediatrics, Sapporo Tokushukai HospitalSapporo, Japan

**Keywords:** pneumonia, cytokine, interleukin-18, autoimmunity, immune complex, vasculitis, vasculopathy, macrolide resistance

## Abstract

The list of extrapulmonary manifestations due to *Mycoplasma pneumoniae* infection can be classified according to the following three possible mechanisms derived from the established biological activity of *M. pneumoniae*; (1) a direct type in which the bacterium is present at the site of inflammation and local inflammatory cytokines induced by the bacterium play an important role (2) an indirect type in which the bacterium is not present at the site of inflammation and immune modulations, such as autoimmunity or formation of immune complexes, play an important role, and (3) a vascular occlusion type in which obstruction of blood flow induced either directly or indirectly by the bacterium plays an important role. Recent studies concerning extrapulmonary manifestations have prompted the author to upgrade the list, including cardiac and aortic thrombi as cardiovascular manifestations; erythema nodosum, cutaneous leukocytoclastic vasculitis, and subcorneal pustular dermatosis as dermatological manifestations; acute cerebellar ataxia, opsoclonus-myoclonus syndrome, and thalamic necrosis as neurological manifestations; pulmonary embolism as a respiratory system manifestation; and renal artery embolism as a urogenital tract manifestation. Continuing nosological confusion on *M. pneumoniae*–induced mucositis (without skin lesions), which may be called *M. pneumoniae*-associated mucositis or *M. pneumoniae*-induced rash and mucositis separately from Stevens-Johnson syndrome, is argued in the dermatological manifestations. Serological methods are recommended for diagnosis because pneumonia or respiratory symptoms are often minimal or even absent in extrapulmonary manifestations due to *M. pneumoniae* infection. Concomitant use of immunomodulators, such as corticosteroids or immunoglobulins with antibiotics effective against *M. pneumoniae*, can be considered as treatment modalities for most severe cases, such as encephalitis. Further studies would be necessary to construct a comprehensive therapeutic strategy, covering microbiology (antibiotics), immunology (immunomodulators), and hematology (anticoagulants). The possible influence of the emergence of macrolide-resistant *M. pneumoniae* on extrapulmonary manifestations, which can be considered of limited clinical threat in Japan where the resistant rate has currently decreased, is discussed on the basis of unique biological characteristics of *M. pneumoniae*, the smallest self-replicating organism.

## Introduction

*Mycoplasma pneumoniae* has been known to cause a wide variety of extrapulmonary diseases, including several organs of the human body, but its pathomechanisms remain largely unknown. Following is a list of extrapulmonary manifestations due to *M. pneumoniae* infection classified according to the three possible pathomechanisms: (1) a direct type in which the bacterium is present at the site of inflammation and local inflammatory cytokines induced by the bacterium play an important role (2) an indirect type in which the bacterium is not present at the site of inflammation and immune modulations, such as autoimmunity or formation of immune complexes, play an important role (3) a vascular occlusion type in which obstruction of blood flow induced either directly or indirectly by the bacterium plays an important role (Narita, 2009, 2010, 2011a). Several years have passed since the initial list was presented and recent studies have prompted the author to upgrade the list (Table 1). While constructing the list, according to the primary policy (Narita, 2009, 2010, 2011a), diseases that can reasonably be considered true extrapulmonary manifestations due to *M. pneumoniae* infection on the basis of established biological ability of *M. pneumoniae* were preferentially selected, although it is hard to prove exactly the causal relation between *M. pneumoniae* infection and the development of diseases in indirect type manifestations. Because more recent studies are preferentially cited in this review, many fundamentally important matters are not mentioned here; frequent absence of pneumonia in the direct type manifestations, cold agglutinins in hematological manifestations, autoantibodies in neurological manifestations, and immunodeficiency in arthritis, among others. Also refer to the previous reviews (Narita, 2009, 2010, 2011a) for further discussions on those matters.

**Table 1:** **Extrapulmonary manifestations due to *M. pneumoniae* infection classified according to the involved pathomechanisms**.

**Manifestations**	**Direct type^a^**	**Indirect type^b^**	**Vascular occlusion type^c^**	**Undetermined^d^**
Cardiovascular system	Pericarditis, Endocarditis	Myocarditis, Kawasaki disease	Cardiac thrombus, Aortic thrombus	
Dermatological		Erythema multiforme, Urticaria, Anaphylactoid purpura, EN, CLV, SJS, MPAM, SPD		
Digestive organ	Early onset hepatitis	Late onset hepatitis	Pancreatitis	
Hematological/Hematopoietic system		Autoimmune hemolytic anemia, Hemophagocytic syndrome, Thrombocytopenic purpura, Infectious mononucleosis	Disseminated intravascular coagulation, Splenic infarct	
Musculoskeletal system	Arthritis			Rhabdomyolysis
Neurological	Early onset encephalitis, Early onset myelitis, Aseptic meningitis	Late onset encephalitis, Late onset myelitis, Guillain-Barré syndrome, Cranial/peripheral neuropathies, Cerebellitis, Acute cerebellar ataxia, Opsoclonus-myoclonus syndrome	Stroke, Psychological disorders, Striatal necrosis, Thalamic necrosis	Acute disseminated encephalomyelitis
Respiratory system			Pulmonary embolism	
Sensory organ	Otitis media	Conjunctivitis, Iritis, Uveitis	Sudden hearing loss	
Urogenital tract		Glomerulonephritis, IgA nephropathy	Priapism, Renal artery embolism	

a *M. pneumoniae causes inflammation at the local site through the induction of cytokines*.

b *M. pneumoniae causes inflammation through immune modulation such as autoimmunity, or formation of immune complexes*.

c *M. pneumoniae causes vasculitic and/or thrombotic vascular occlusion with or without systemic hypercoagulable state*.

d *Either or all of the above three types of mechanisms may be involved*.

*Underlines indicate diseases which are newly included in the panel or grouped into a different type of manifestation from the previous list (Narita, 2010)*.

*EN, erythema nodosum; CLV, cutaneous leukocytoclastic vasculitis; SJS, Stevens-Johnson syndrome; MPAM, Mycoplasma pneumoniae-associated mucositis; SPD, Subcorneal pustular dermatosis*.

### Cardiovascular system manifestations

Cardiac thrombi in the left atrium (Bakshi et al., 2006), in the right ventricle (Nagashima et al., 2010), and an aortic thrombus (Flateau et al., 2013) have been reported as the vascular occlusion type manifestation of the cardiovascular system. Interestingly, all the cases revealed the existence of some type of antiphospholipid antibodies in the blood, such as anticardiolipin antibody and lupus anticoagulant, which can be raised during *M. pneumoniae* infection through molecular mimicry between *M. pneumoniae* cell components and human phospholipids (Narita, 2011a). A mechanism speculating about how these antibodies modulate the coagulation system leading to thrombosis is incompletely understood. These antibodies in most cases disappear during convalescence and the hypercoagulable state does not last for many months. Pneumonia may or may not be present. A short comprehensive review on this topic is presented in (Flateau et al., 2013).

While Kawasaki disease associated with *M. pneumoniae* infection is not unusual in Japan (Narita, 2010, 2011a) and may be found in Korea (Lee et al., 2011), the disease association is rarely reported outside Asia; however, few recent cases were observed in Italy (Vitale et al., 2010) and the United States (Ebrahim et al., 2011). Considering that pneumonia is not a hallmark of mycoplasmal infection, further surveys outside Asia would more precisely delineate the occurrence of this disease association among different ethnic groups. A short analytical review on this topic can be found in (Lee et al., 2011).

A recent report from China on myocardial damages during *M. pneumoniae* infection presented a little evidence for some type of immune modulation by *M. pneumoniae* (Fan et al., 2015).

### Dermatological manifestations

Erythema nodosum, which is considered to be an immune-mediated disease, mainly affects young women (< 30 years old) and is characterized clinically by tender erythematous nodules (diameter > 1 cm) on lower legs and histologically by septal panniculitis (Cribier et al., 1998; Kakourou et al., 2001). While its frequency among mycoplasmal infections has been reported to be rather small, that is, in 3/27 (11%) patients with established etiology (Kakourou et al., 2001) or 1/32 (3.1%) patients undergoing mycoplasmal serology testing (Cribier et al., 1998), increasing awareness of the disease association (Kano et al., 2007; Schalock and Dinulos, 2009; Shimizu et al., 2012) allows it to become a subject of specific reviews (Greco et al., 2015; Terraneo et al., 2015). This disease must be included in the indirect type manifestations. Pneumonia is infrequent in this disease.

Cutaneous leukocytoclastic vasculitis is a pathological entity of skin disease characterized histologically by a neutrophilic perivascular infiltrate and clinically by erythematous macropapular rash mainly on lower extremities; it resembles erythema nodosum but is less tender and smaller in size (Kakourou et al., 2001). Several cases of this disease have been reported in association with *M. pneumoniae* infection (Van Bever et al., 1992; Perez et al., 1997; Perez and Montes, 2002; Greco et al., 2007; Trčko et al., 2012; Lee et al., 2015; Terraneo et al., 2015). Interestingly the reported cases were almost always accompanied by other organ involvement such as glomerulonephritis (Lee et al., 2015), arthritis (Perez et al., 1997; Lee et al., 2015), or arthralgia (Trčko et al., 2012), retinal vasculitis (Greco et al., 2007), encephalitis (Perez and Montes, 2002), and acute respiratory distress syndrome, erythema multiforme, and pancreatitis (Van Bever et al., 1992). Because circulating immune complexes are considered to play a critical role in the pathogenesis of cutaneous leukocytoclastic vasculitis (Van Bever et al., 1992; Perez and Montes, 2002; Trčko et al., 2012), this must be a partial manifestation of systemic vasculitic disease as a consequence of immune dysregulation elicited by an *M. pneumoniae* infection. Pneumonia may or may not be present.

Nosological confusion still exists concerning the spectrum of skin and mucous membrane diseases, including Stevens-Johnson syndrome (SJS), Fuchs syndrome, toxic epidermal necrolysis, and erythema multiforme major (Schalock and Dinulos, 2009; Wetter and Camilleri, 2010; Kunimi et al., 2011; Meyer Sauteur et al., 2012; Canavan et al., 2015a; Vujic et al., 2015). It has been acknowledged that *M. pneumoniae* is the most frequent infectious agent identified in “typical SJS” (Schalock and Dinulos, 2009; Wetter and Camilleri, 2010; Kunimi et al., 2011), presenting with fever, conjunctivitis, stomatitis, generalized, often bullous cutaneous lesions (macules and flat atypical target lesions) involving < 10% of body surface, and severe morbidity and substantial mortality (Meyer Sauteur et al., 2012). Moreover, an outbreak of *M. pneumoniae*-associated “typical SJS” was recently reported (Olson et al., 2015). In parallel with this, presence of another distinct form of diseases mimicking SJS and without skin lesions, is widely noticed and is often called as “atypical SJS” or “incomplete SJS.” Although universal agreement has not yet been established, those can be a distinct entity and are presently called *M. pneumoniae*-associated mucositis (Schalock and Dinulos, 2009; Meyer Sauteur et al., 2012; Vujic et al., 2015) or *M. pneumoniae*-induced rash and mucositis (Canavan et al., 2015a,b; Norton, 2015). From a clinical point of view, *M. pneumoniae*-associated mucous membrane diseases, irrespective of whether they are “typical” or “atypical,” have been considered less severe, often sensitive to corticosteroid therapy, and with fundamentally good prognosis when compared with drug-induced diseases (Schalock and Dinulos, 2009; Wetter and Camilleri, 2010; Kunimi et al., 2011; Meyer Sauteur et al., 2012; Canavan et al., 2015a; Vujic et al., 2015). These factors may be important especially for physicians in treating this spectrum of diseases, because to distinguish *M. pneumoniae*-associated diseases from drug-induced diseases early in the course allows them to predict the better prognosis for the *M. pneumoniae*-associated diseases. One factor which favors the *M. pneumoniae*-associated rather than the drug-induced diseases is younger age as it occurs more often in children and younger adults (Wetter and Camilleri, 2010; Kunimi et al., 2011; Canavan et al., 2015b; Norton, 2015). Meanwhile, three independent reports have similarly pointed out that severe ocular lesions were fairly frequent in the *M. pneumoniae*-associated diseases compared to the drug-induced diseases (Wetter and Camilleri, 2010; Kunimi et al., 2011; Olson et al., 2015), and might be an additional diagnostic indicator. Regarding pathogenesis, immunological mechanisms such as autoimmunity and immune complex-mediated vascular injury have been suspected irrespective of whether it is *M. pneumoniae*-associated or not. Some authors have speculated that the synergistic effects of *M. pneumoniae* infection (and ensuing immune dysregulation) and drug exposure are important factors in developing mucous lesions (Schalock and Dinulos, 2009; Shimizu et al., 2012; Kurata et al., 2016). The reason why the *M. pneumoniae*-associated lesions are confined to the mucous membranes remains unclear. Lastly, the fact that *M. pneumoniae* was isolated from skin blister fluid on at least two independent occasions must not be ignored, which suggests the possibility of a direct type mechanism (Lyell et al., 1967; Meseguer et al., 1986). Because *M. pneumoniae* can never infect squamous cell epithelium, hematogenous transfer of *M. pneumoniae* from the respiratory tract to the skin might generate the inflammatory bullous lesions through the induction of cytokines.

Although very rare, subcorneal pustular dermatosis must be associated with *M. pneumoniae* infection (Lombart, 2014; Bohelay et al., 2015), which can be considered an indirect manifestation.

### Digestive organ manifestations

A report on liver dysfunction in adults further substantiated the premise that hepatitis can be grouped into the two categories, consisting of the early- and late-onset types, the former being reported to occur at a median of 4 days from the respiratory onset and the latter at 13 days (Shin et al., 2012). Molecular mimicry between mycoplasmal cell components and sialo-oligosaccharides displayed on hepatic cell surfaces was speculated as a pathomechanism for the late-onset type. Another recent report on hepatic damages during *M. pneumoniae* infection also presented a little evidence for some type of immune modulation by *M. pneumoniae* (Fan et al., 2015).

A case report on necrotizing pancreatitis (Yang et al., 2015) favors vascular occlusion as the etiology of acute pancreatitis associated with *M. pneumoniae* infection as previously suggested (Van Bever et al., 1992; Narita, 2010).

### Hematological/hematopoietic system manifestations

A case of splenic artery embolism was reported as a vascular occlusion type manifestation (Flateau et al., 2013).

### Musculoskeletal system manifestations

We have reported that production of tumor necrosis factor-α might play a role in the pathogenesis of rhabdomyolysis associated with *M. pneumoniae* infection (Oishi et al., 2012).

### Neurological manifestations

Opsoclonus-myoclonus syndrome is a rare neurological disorder characterized by involuntary, irregular, and multidirectional eye movements with myoclonus predominantly affecting the head and trunk and signs of cerebellar ataxia, especially the inability to stand and walk. While this syndrome has been known to occur in association with neuroblastomas in infants between 6 and 36 months and with various types of malignant tumors in adults, infectious etiologies are also known. Recent studies on this syndrome in association with *M. pneumoniae* infection in children (Huber et al., 2010; Shiihara and Takahashi, 2010) as well as in adults (Mesraoua et al., 2011; Nunes et al., 2011), which have suggested immune pathogenesis, have made it reasonable to assume that this syndrome can be included in the indirect type manifestations.

In addition to striatal necrosis (Narita, 2009, 2011a), brain diseases, in which characteristic bilateral lesions are observed on neuroimaging, have not infrequently been reported in association with *M. pneumoniae* infection. The affected areas include the pons (Perez and Montes, 2002), thalamus (Ashtekar et al., 2003), basal ganglia, and thalamus (Fusco et al., 2010), striatum and brain stem (Bae et al., 2011), and splenium of corpus callosum (Shibuya et al., 2012). Vasculitic necrosis is considered a presumptive etiology in some (Perez and Montes, 2002; Ashtekar et al., 2003), and immune pathogenesis is considered in others (Fusco et al., 2010; Bae et al., 2011). In this context, the clinical picture of a case presented in Perez and Montes (2002) fairly resembles that of acute necrotizing encephalopathy, which favors vasculitic etiology (Narita, 2002), whereas the intrathecal production of interleukins-6 and -8 was found in the patients with striatal necrosis (Yuan et al., 2015), as reported in the cases of encephalitis (Narita et al., 2005), which favors immune pathogenesis. A recent comprehensive study suggested that these manifestations are not peculiar but is a common form of encephalitis in children (Al-Zaidy et al., 2015). Representative of these diseases, both striatal and thalamic necrosis are included in the list of this review as vascular occlusion type manifestations. Meanwhile, some nosological alterations might be necessary concerning the use of the term “necrosis” for the fundamentally benign, reversible lesions, which are a characteristic of *M. pneumoniae* infection (Fusco et al., 2010).

Concerning disseminated encephalomyelitis, a recent paper reported that the genome of *M. pneumoniae* was detected in cerebrospinal fluid, suggesting the direct type mechanism (Matsumoto et al., 2009), whereas another paper reported a dramatic improvement after plasma exchange, suggesting the indirect type mechanism leading to vasculopathy (Gupta et al., 2009). The aforementioned study has suggested that a single etiology cannot explain the pathogenesis of acute disseminated encephalomyelitis (Al-Zaidy et al., 2015). In addition, the study also suggested that the classification for encephalitis can also be applied to transverse myelitis (the early onset, direct type or the late onset, indirect type).

Two cases of transient Parkinsonism in association with *M. pneumoniae* infections have recently been reported (Tay et al., 2014). With more attention given to *M. pneumoniae* infections even in the absence of respiratory symptoms, more cases of *M. pneumoniae* infection-associated psychological disorders would be diagnosed especially when it is transient and occurs in children or young adults.

Cerebellitis has been constantly reported as part of a disease involving multiple parts of the brain (Christo et al., 2010; Bae et al., 2011; Meyer Sauteur et al., 2014a) or as an isolated disease (Shkalim et al., 2009; Simpkins et al., 2012; Schmucker et al., 2014). Immune-mediated pathogenesis has been advocated for cerebellitis and is most likely an indirect type manifestation. The fact that lymphocytic infiltration was found in the cerebellar tissue in the late onset case (Simpkins et al., 2012) is of some interest because the neutrophilic infiltration has typically been found in the cerebral tissues of early onset cases (Bruch et al., 2001; Stamm et al., 2008). Implication of these observations on pathogenesis remains unclear, but accumulation of histological investigations should provide us with clues for further understanding of the pathogenesis of neurological manifestations.

In acute cerebellar ataxia the evidence of neural inflammation is apparently absent and an immune-mediated pathogenesis has been suggested (Cimolai et al., 1994); this has been added to the list separately from cerebellitis.

Although it is still premature to conclude that it is a true manifestation of *M. pneumoniae* infection, a peculiar and rather pathologically identified disease entity called “mycoplasmal cerebral vasculopathy” has recently been suggested (Zu-Rhein et al., 2007; Ferreira, 2011; Rhodes et al., 2011). Because of its slowly progressive clinical course that occurs over several years with episodic encephalopathy and movement disorders, it is hard to determine whether the disease constitutes a distinct clinical entity. Nevertheless, the findings that *M. pneumoniae* antigens were found in the cytoplasm of brain microvascular endothelial cells as well as in microvascular lumina by histological investigations are highly interesting. This appears to further support the ability of hematogenous transfer of *M. pneumoniae* to the brain and to elicit vasculitis or vasculopathy at those local sites.

**Figure 1:**
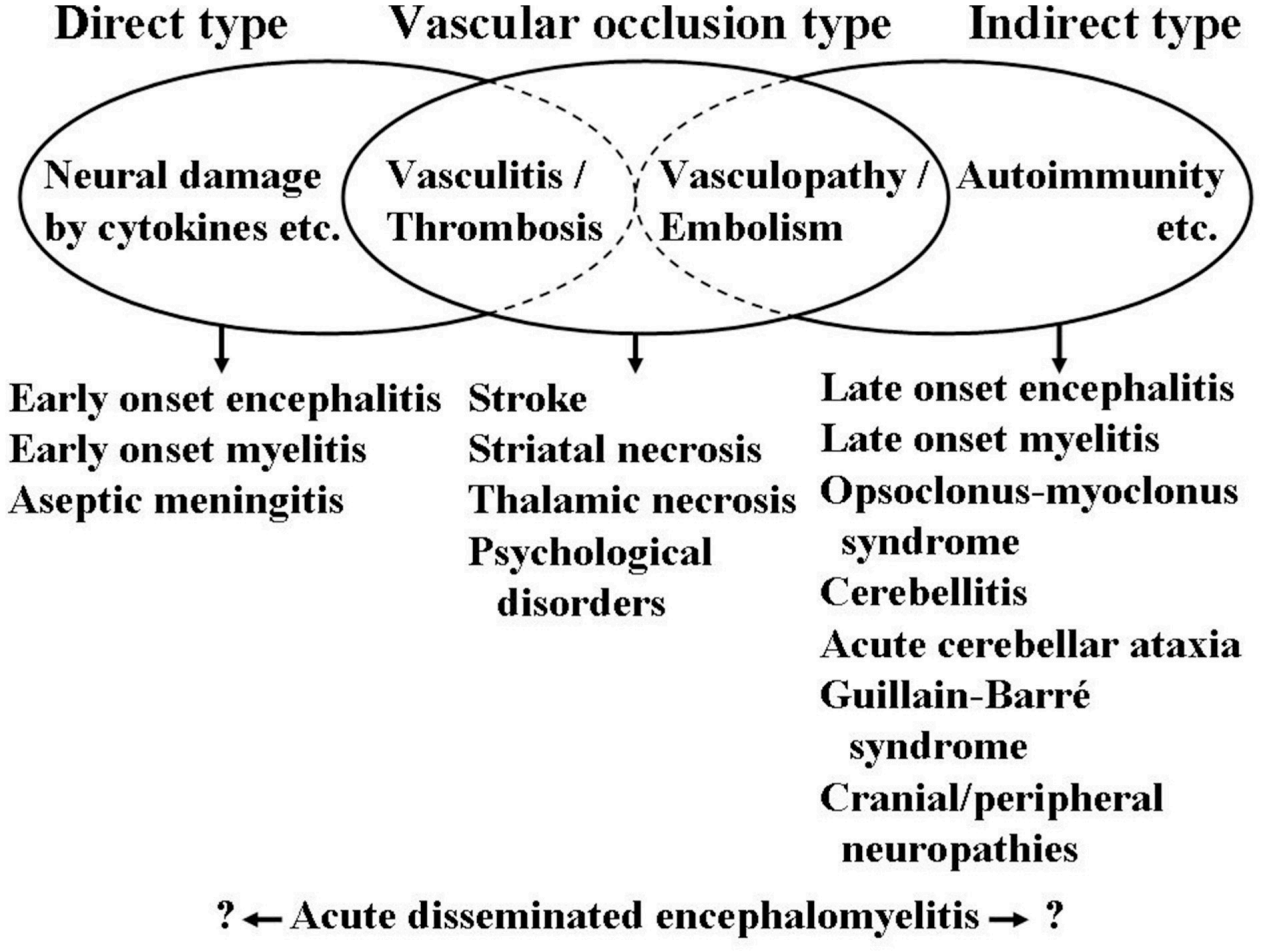
**Schematic presentation of neurological manifestations due to *M. pneumoniae* infection classified according to the possible pathomechanisms**. Cytokines involving interleukin-6 and interleukin-8, among others. Vasculitis, thrombosis, vasculopathy, and embolism involving formation of immune complexes, production of antiphospholipid antibodies, among others. Autoimmunity involving molecular mimicry between mycoplasmal cell components and gangliosides, and galactocerebrosides, among others. These mechanisms are not mutually exclusive and can concomitantly work in a patient resulting in perplexed clinical features of neurological manifestations.

At the end of this section, a schematic presentation is shown in Figure 1 for the neurological manifestations due to *M. pneumoniae* infection classified according to the possible pathomechanisms, which can be perplexing and sometimes overlapping. This figure suggests one possibility and is amenable to improvement by future studies. More information on neurological manifestations is presented in recent studies (Bitnun and Richardson, 2010; Meyer Sauteur et al., 2014b).

### Respiratory system manifestations

A few cases of pulmonary embolism have been reported in association with *M. pneumoniae* infection (Sterner and Biberfeld, 1969; Brown et al., 2008; Graw-Panzer et al., 2009). Production of antiphospholipid antibodies has been shown to be an underlying mechanism for thrombus formation (Brown et al., 2008; Graw-Panzer et al., 2009).

### Urogenital tract manifestations

Each case of glomerulonephritis (Shimizu et al., 2012) or glomerulonephritis with interstitial nephritis (Lee et al., 2015) was recently reported along with multiple skin lesions mentioned in the previous dermatological section. The genome of *M. pneumoniae* was detected by polymerase chain reaction in a serum sample from a patient with glomerulonephritis (Chen et al., 2015), which further substantiated the assumption that circulating immune complexes containing mycoplasmal cell components are probably involved in the pathogenesis.

A case of renal artery embolism was reported in a patient with multiple embolisms (Flateau et al., 2013).

A new case of pediatric priapism was recently reported (Jacobs et al., 2015), which substantiated that this condition can be an extremely rare but reasonable vascular occlusion type manifestation.

## Diagnosis of extrapulmonary manifestations due to *M. pneumoniae* infections

Since the primary site of infection and subsequent propagation of *M. pneumoniae* is restricted to the ciliated epithelium of the lower respiratory tract, any existing bacterial cells cannot be transferred to the upper respiratory tract in the absence of strong cough. In this context, as repeatedly mentioned in this and the previous reviews (Narita, 2009, 2010, 2011a), extrapulmonary manifestations due to *M. pneumoniae* infection often occur in the absence of pneumonia or even in the absence of respiratory symptoms. For this reason, molecular detection or culture methods using routine clinical samples obtained from the upper respiratory tract (such as pharyngeal swabs) are not always adequate for diagnosing extrapulmonary manifestations. Therefore, the diagnosis should be done by serological methods, which usually requires obtaining a second serum sample. On a few special occasions, molecular detection methods may be applied for non-respiratory samples, such as cerebrospinal fluid for encephalitis. For cases with abundant cough, point-of-care tests, which have recently been developed in Japan and include the loop-mediated isothermal amplification method (Kakuya et al., 2014; Petrone et al., 2015) or the antigen detection method (Miyashita et al., 2015b), should be used because they may help in rapid diagnosis during the acute phase.

## Treatment of extrapulmonary manifestations due to *M. pneumoniae* infection

There is no doubt that aberrant host immune responses play a critical role in the development of extrapulmonary manifestations due to *M. pneumoniae* infections. Therefore, immunomodulators, such as corticosteroids or immunoglobulins, should be beneficial for the most severe cases, such as encephalitis or SJS. Moreover, anticoagulation therapy should be highly promising for the vascular occlusion type manifestations. In any of the cases, antibiotics effective against *M. pneumoniae* must be used concomitantly to reduce the amount of *M. pneumoniae* cells in the respiratory tract; this consequently results in the reduction of excessive antigenic stimuli. Larger studies would be necessary to construct the comprehensive therapeutic strategy covering microbiology (antibiotics), immunology (immunomodulators), and hematology (anticoagulants).

## Possible influence of the emergence of macrolide-resistant *M. pneumoniae* on extrapulmonary manifestations

Since 2000, when the first case of pneumonia due to macrolide-resistant *M. pneumoniae* was diagnosed in Japan (Okazaki et al., 2001), macrolide resistance has grown to be a significant problem in some countries, particularly in eastern Asia. A few cases of extrapulmonary manifestations due to macrolide-resistant *M. pneumoniae* have been reported (Atkinson et al., 2011; Koga et al., 2012; Oishi et al., 2012; Shen et al., 2013; Zhou et al., 2014). While the studies from China have reported that a substantial number of complications (most frequently liver and myocardial dysfunctions) occurred in patients infected by resistant strains (Shen et al., 2013; Zhou et al., 2014), no appreciable increase in the number of extrapulmonary manifestations has been observed in Japan in conjunction with a significant increase in the number of pneumonia patients infected by the resistant strains. This must be in part due to the impairment of the growth ability of resistant strains of *M. pneumoniae* when compared with the sensitive strains (Ohya et al., 2009, 2010; Pauchant et al., 2009) Some reasons for this are described in the following paragraphs.

*M. pneumoniae* is one of the smallest self-replicating organisms and has many peculiar biological characteristics. First of all, the most important characteristic associated with drug resistance is that extrinsic genes, such as plasmids or transposons, do not function within *M. pneumoniae* cells under natural conditions (Bébéar and Pereyre, 2005). Consequently, the resistant mechanism of *M. pneumoniae* is exclusively due to a point mutation in the domain V of 23S rRNA. Second, since *M. pneumoniae* has only one operon for constructing ribosomes (Himmelreich et al., 1996), the resistant strains that harbor a point mutation within their ribosome genes are exclusively mutants of ribosomes. Therefore, they suffer from less efficient protein synthesis and are deficient in growth ability (Narita, 2011b). In fact, no excessive morbidity has been observed particularly ascribed to the drug resistance in the reported cases (Atkinson et al., 2011; Koga et al., 2012; Oishi et al., 2012). In addition to that, serum levels of IL-18, which represent disease activity of *M. pneumoniae* infection (Narita et al., 2000; Tanaka et al., 2002; Oishi et al., 2011; Miyashita et al., 2015a), were rather lower in patients with pneumonia infected by the resistant strains than in patients infected by the sensitive strains (Matsuda et al., 2013). Taken together, the emergence of macrolide-resistant *M. pneumoniae* must not be a significant clinical threat concerning extrapulmonary manifestations of *M. pneumoniae* infection at least in Japan, where the resistant rate has fundamentally been decreasing according to the current data obtained from 2013 to 2015 (presented at domestic Japanese meetings).

## Author contributions

The author confirms being the sole contributor of this work and approved it for publication.

### Conflict of interest statement

The author declares that the research was conducted in the absence of any commercial or financial relationships that could be construed as a potential conflict of interest.
